# Private provider practices and incentives for hypertension management in rural and peri-urban Telangana, India– a qualitative study

**DOI:** 10.1186/s12913-024-11560-5

**Published:** 2024-10-09

**Authors:** Samriddhi S Gupte, Ashish Sachdeva, Aman Kabra, Bhanu Pratap Singh, Ashish Krishna, Anupam Khungar Pathni, Bhawna Sharma, Andrew Moran, Amarendar Reddy Mamindla, Nanda Kishore Kannuri, Sarang Deo

**Affiliations:** 1 Indian School of Business, Hyderabad, India; 2 Pennsylvania State University, University Park, PA USA; 3 Resolve to Save Lives, New Delhi, India; 4 Independent Consultant, New Delhi, India; 5 Resolve to Save Lives, New York, NY USA; 6 Division of General Medicine, Columbia University Irving Medical Center, New York, NY USA; 7 Indian Institute of Public Health, Hyderabad, India; 8 University of Hyderabad, Hyderabad, India; 9 Indian Institute of Management, Udaipur, India

**Keywords:** Private providers, Hypertension, Qualitative design, Hypertension management practices

## Abstract

Hypertension poses a critical threat to health in India, being the predominant risk factor for mortality and disability. With over 70% of outpatient care being provided by the private sector, our study investigated the practices and incentives of private health providers in screening, diagnosing, treating, monitoring, and counseling hypertension patients in rural and peri-urban India. Conducted from April 2020 to February 2021, the qualitative study involved 46 participants, including various healthcare professionals and patients in three Telangana state districts. Analysis revealed a lack of recognition of hypertension’s gravity among private providers in these areas, leading to inconsistent screening practices and varied diagnostic thresholds, particularly among rural medical practitioners. Both formal and informal providers lacked standardized protocols and follow-up mechanisms, with limited technical knowledge about hypertension observed, especially among rural practitioners who were often the first point of contact. Drug effectiveness, and incentives from pharmaceutical sales representatives influenced prescribing practices. Diagnostic labs also offered financial incentives for patient referrals. Thus, aligning providers’ objectives with patient preferences and public health goals is crucial. To encourage evidence-based hypertension care, the government and NGOs could implement strategies such as tailored incentives, financial rewards, tax benefits, accreditation, and recognition for private healthcare providers. Professional bodies in the private sector should establish programs emphasizing quality assurance and certifications. Future research should focus on designing and testing new models for private sector hypertension service delivery, coupled with targeted interventions to enhance care in rural and peri-urban settings.

**Supplementary Information:**

The online version contains supplementary material available at 10.1186/s12913-024-11560-5.

## Introduction

Over the past two decades, India has experienced an epidemiological shift from infectious to non-communicable diseases [1]. Cardiovascular diseases (CVDs) have become a leading cause of mortality, significantly affecting Indians during their most productive years [2–4]. In 2019, CVDs accounted for 35.5% of disability-adjusted life years lost in India [5], compared to 26.8% globally [6]. Hypertension, a major CVD risk factor, caused an estimated 10.8 million deaths worldwide in 2019 [7]. Its prevalence is rising, projected to reach nearly 30% globally by 2025 from the current 26% [8]. In 2017-18, 28.3% of Indian adults had elevated blood pressure [9], burdening the healthcare system. Rapid urbanization has increased CVDs in urban, rural, and peri-urban areas, where hypertension risk is rising [10, 11]. Peri-urban zones, between rural and urban settings, face challenges like poor health literacy and pollution [8]. Smaller studies show rural hypertension prevalence at 15–20% [12, 13]. The National Non-communicable Monitoring Survey reported 25.7% of rural adults with elevated blood pressure in 2017-18.

India’s healthcare system is primarily led by the private sector, which delivers over 70% of outpatient care [14–16]. This sector includes allopathic doctors, AYUSH providers, and informal practitioners. The demand for private healthcare has surged due to the rising burden of non-communicable diseases [17]. The private sector encompasses 93% of hospitals, 80–85% of doctors, and 70% of outpatient visits [13, 14]. Economic and non-economic incentives influence private providers’ behavior [18], but their models often lack provisions for ongoing care of chronic conditions like hypertension [19, 20].

We aimed to understand private providers’ practices and incentives in rural and peri-urban areas for diagnosing and managing hypertension. The study will aid in designing targeted interventions for better hypertension management in these regions.

## Data and methods

###  Study design

We conducted a descriptive qualitative study to understand the practices and incentives for private providers for hypertension care and management. The study was conducted in three districts of Telangana, India: Warangal Urban, Karimnagar, and Sircilla from April 2020 to February 2021.

### Study setting

In Telangana, most people access private healthcare. In rural Telangana, 41% of patients used private hospitals, and 37% visited private clinics [14]. Allopathy is the most sought-after system of medicine [21]. Warangal Urban has a 68.58% urban population, Sircilla is mainly rural with two urban towns, and Karimnagar is 85% urban. According to NFHS 5 (2019-20), elevated blood pressure (systolic ≥ 140 mm Hg and/or diastolic ≥ 90 mm Hg) was found in 33% of men and 25% of women in Warangal, 36% of men and 30.2% of women in Sircilla, and 35% of men and 30.2% of women in Karimnagar.

### Study participants

We used a purposive sampling technique, followed by snowball sampling to recruit participants for the study i.e. we reached out to one participant from each group and then they assisted us in recruiting participants from their peers. We interviewed a total of 46 participants (Interview guides: Supplementary file 1). To gather a comprehensive perspective, we interviewed different types of providers and patients. We defined private providers as healthcare practitioners who operate independently and are not employed by the central or state government. This category included three types of private providers:

- Modern medicine providers (*n* = 5), which comprise certified allopathic doctors (Bachelor of Medicine and Bachelor of Surgery or above) recognized by the medical board and licensed to practice medicine. Further referred to as MMPs throughout the study.
- AYUSH practitioners (*n* = 2), who are non-allopathic doctors licensed to practice biomedicines by their respective regulatory authority and specialize in Ayurveda, Yoga, Unani, Siddha, or Homeopathy.
- Informal care providers, specifically rural medical practitioners (RMPs) (*n* = 10), who do not undergo formal training at recognized institutions but provide informal care.

Additionally, we interviewed other stakeholders in the study, such as pharmacists (*n* = 04), pharmaceutical sales representatives (*n* = 02), and diagnostic laboratory managers (*n* = 02), to understand their relationship with the providers and their role in hypertension care. Patient (*n* = 21) interviews served as a means of triangulating the information obtained from the providers.

The study protocol number (ISB-IRB 2020-08) was approved by the Institutional Review Board of the Indian School of Business (Hyderabad, India). Prior to the interview, participants were informed about the study, and written consent was obtained in the local language.

### Data collection

We developed semi-structured qualitative interview guides based on the themes identified from the literature. The interview guides aimed to capture essential aspects of the patient care pathway, including the sequence of touch points, frequency of visits, patient movement between stakeholders, services provided by each stakeholder, referral practices, and the incentives received by private providers for hypertension care and management. These guides were piloted in locations near Hyderabad. These locations were different than the study sites.

Subsequently, we conducted in-depth, face-to-face interviews using the interview guides. The interviews were conducted in the local language, Telugu, and were recorded for accuracy. Later, the interviews were transcribed verbatim and translated into English by a bilingual author. To ensure the accuracy of translations, another bilingual author, whose native language is Telugu, verified the translated content.

### Data analysis

We opted for deductive thematic analysis for its advantages in conducting exploratory research, a strategy that has been used in multiple studies in the healthcare research domain [22]. To ensure data validity, we employed the member-checking strategy and triangulated the responses from providers and stakeholders with those from patients.

Initially, we familiarized ourselves with the transcribed data by reading it multiple times. We then indexed the data, aligning relevant excerpts with the a priori themes identified from the literature during the design of the interview guides. During this process, we observed the indexed data in its entirety and rearranged, combined, or collapsed textual data, into themes, and sub-themes based on the study’s focus, the data itself, and emerging patterns. This approach enabled us to construct a coherent narrative and address the research question during the mapping and interpretation stage of thematic analysis [23].

In addition to the thematic analysis, we incorporated a framework based on the Chronic Care Model (CCM) to guide our analysis and present our results. The CCM has been widely employed to enhance ambulatory care, operating on the premise that linking informed and activated patient communities with proactive and prepared healthcare teams within a well-organized health system can improve the quality of care [24]. CCM has two broad domains, Health facility level and Community level. The health facility level domain has four sub-domains namely, Delivery System Design, Self-management support, Decision support, and Clinical information system. These are discussed below:

- *Delivery System Design (DSD)* [25]: DSD involves delivering efficient care (screening, diagnosis, treatment) using all team members appropriately, referrals, and patient-centric case management.
- *Self-management Support* [25]: CCM promotes informed, engaged patients managing hypertension daily. Key activities at the facility include counseling on lifestyle, diet, exercise, and home monitoring.
- *Decision Support* [25]: Promotes clinical care aligned with scientific evidence and patient preferences, ensuring quality care through provider education.
- *Clinical Information System (CIS)* [25]: Organizes patient and population data for effective care. Key components include adherence, patient monitoring, and maintaining medical records.

## Results

### Screening and diagnosis

Routine opportunistic hypertension screening was uncommon among providers, who checked blood pressure only when symptoms like dizziness appeared. Providers reported checking blood pressure at least twice before diagnosing hypertension, but the intervals varied. Two out of five MMPs waited a week between checks, while one provider took multiple readings in a single day, confirming hypertension if readings exceeded 140/90 mmHg.

*“If the patient has symptoms*,* I take 2–3 readings on the same day and if the readings are 140/90 or above then I confirm hypertension.”- [MMP, Warangal*].

Of the 10 RMPs interviewed, six waited a week after an initial high blood pressure reading before diagnosing hypertension and referring patients to an MMP. The remaining RMPs reported intervals of two to four days between readings.

*“I confirm hypertension after measuring BP daily in the morning and evening for 2 days.*” – [RMP, Karimnagar].

AYUSH providers also showed variation: one took multiple blood pressure readings on the same day to confirm hypertension, while the other observed the patient for a week.

“*I measure BP 2–3 times for new patients on the same day*.” – [AYUSH, 35, Warangal].

Among the 17 providers interviewed, four considered a blood pressure reading of 150/90 mmHg as indicative of hypertension, while six used the standard threshold of 140/90 mmHg. One AYUSH provider applied varying thresholds, and four providers considered age in their hypertension diagnosis.

*“In my view*,* the B.P. threshold is for diastolic 90 and systolic 90 plus his/her age. For example*,* if a person’s age is 40 then 90 + 40 = 130 is the B.P. threshold.” –[AYUSH*,* Karimnagar].*

*“150 is the B.P. threshold reading to diagnose hypertension at my level.”* – *[MMP*,* Warangal].*

Most providers used mercury sphygmomanometers in clinics, while RMPs preferred digital devices during home visits. This practice was consistent among all providers.

Patients confirmed being screened for hypertension when symptomatic and reported variability in the frequency of blood pressure measurements before diagnosis.

*“I used to have accelerated heartbeat (palpitations)*,* and uneasiness. I visited hospital at Karimnagar because of it and there the doctor tested and found hypertension.” – [43*,* M]*.

*“The doctor checked my blood pressure 2–3 times in same day. It was 140 he said.” – [71*,*M]*.

*“After taking 2 readings in one month the doctor confirmed that I had hypertension.” – [46*,*M]*.

### Treatment

Treatment practices varied significantly. Homeopathy and Ayurveda practitioners used their formulations, while half of the 10 RMPs focused on lifestyle counseling and referrals to MMPs. The other RMPs prescribed branded medications.

*“… (I) suggest irbesartan tablets and irbesartan H to 60 + age group…In normal conditions*,* amlodipine 5 mg or 2.5 mg is given according to their BP level and body condition.”* – [*RMP*,* Warangal].*

“*We prescribe medicine like Arjuna Vati*,* Sarpaganda*,* Lashuna kshetra pakam (Ayurvedic medicines)*,* etc tablets or Rasayana is given to patients.” –[AYUSH*,* Karimnagar]*.

MMPs tailored prescriptions based on molecular formulations or combinations, starting with low doses and adjusting if patients did not respond adequately.

*“…for beginning*,* amlodipine 5 mg is given. Initially*,* we start with single molecule but according to the problem like heart issues along with BP.*,* we give double/combination medicine. Generally*,* Telma 40*,* metoprolol is used.”* – [*MMP*,* Warangal]*.

According to a pharmaceutical sales representative, MMPs considered prescribing a particular drug on two factors: effectiveness and the incentives associated with the drugs.

*“The performance (effectiveness) of the drug of a particular brand will influence a doctor’s choice in prescribing a specific brand. Further*,* incentives will also influence the doctor’s choices while prescribing the brand*.” – *[Sales Rep 2]*.

Responses from the patient interviews also concur with the above findings.

“*A few months ago*, *due to my increased BP*,* the doctor increased the dosage… The medicines are certified and branded as per the doctor prescription.”- [52*,* M]*.

*“Earlier I use to consume xxx (Metoprolol) 50 mg but later on doctor has changed it to xxx.( Telmisartan and Metoprolol Succinate combination)” – [51*,* M]*.

### Task allocation/Team structure

All MMPs employed staff, including doctors, lab technicians, and pharmacists. Due to financial constraints, RMPs had no staff, while AYUSH providers had an assistant.

“*No other staff. I only treat the patients and maintain the clinic as well. We can’t afford staff with such less amount as a fee.*” – *[RMP*,* Karimnagar].*

“*Total of 9 members are working in the clinic. They are duty doctors*,* nurses*,* pharmacists*,* lab technicians*,* and other training staff… duty doctor attends to hospitalized patients*,* nurses assist in medication and treatment services*,* a lab test will be done by lab technicians and the pharmacist dispenses medicines”* – *[MMP*,* Warangal]*.

### Referrals and incentives

RMPs referred patients to MMPs, often receiving incentives in cash, kind, or free medicine samples. Incentives depended on diagnostic tests and hospital admissions. Some RMPs were reimbursed for travel by patients. MMPs received positive reinforcements from pharmaceutical companies. Pharmacies were also incentivized for higher sales.

It is important to note that while incentives were present for referrals, there were no specific incentives related to hypertension referrals.

“*No referral between RMPs. We do send patients to a general physician.”* – [*RMP*,* Warangal].*

“*Yes*,* RMPs send patients to our hospital. Generally*,* around 10 patients come in a week…*” – [*Modern medicine provider*,* Warangal].*

“*There are some monetary incentives for a referral by a typical RMP to a GP or super specialist and it depends on tests like ECG*,* 2D-echo*,* hospitalization*,* etc. Sometimes they give gifts like wall clocks*,* medicines*,* newspaper subscriptions*,* B.P monitor*, etc.,* for the clinic.*” – [*RMP*,* Warangal]*.

*“…pharma companies do not provide positive reinforcement to RMPs*,* they only give some medical samples. They give positive reinforcement for doctors/super specialists…” –[RMP*,* Warangal].*

*“Yes*,* doctors get gifts from pharma companies to prescribe their medicines.” –[Pharmacy*,* Karimnagar]*.

*“We provide incentives/gifts and sometimes tour packages are also arranged for the pharmacies that are higher volume sellers compared to others.”-*[Sales Rep 2].

Responses from patient interviews also reveal referrals from RMPs to MMPs.

*“Initially I approached local RMP doctor then he has suggested MD specialist*.” – [60,*M]*.

Diagnostic labs played a role in hypertension management after initial screening. Patients with elevated blood pressure were referred for tests like ECG, kidney function, and lipid profiles. Providers without in-house labs referred patients to nearby labs with established relationships.

*“Mostly the doctors in this area refer their patients to specific laboratories”.-[Lab owner*,* Karimnagar]*.

RMPs also referred patients regularly to the diagnostic labs for similar tests.

*“Few of the patients (3–4 patients) come from RMPs in a week.” –[Lab owner*,* Karimnagar]*.

For the referrals, the laboratories provided incentives to the providers, both for the MMPs and RMPs.

*“For the referral*,* we give 40% to the RMP/GP from the charges”.– [Lab owner*,* Karimnagar]*.

### Patient centric counseling

MMPs spent 10–15 min educating patients on hypertension, lifestyle management, and medication adherence. Some had staff for counseling. All emphasized reducing salt intake, followed by eating non-fatty, non-spicy, and fiber-rich foods, avoiding alcohol and tobacco, and encouraging regular exercise.

“*I spend on an average 10–15 minutes on lifestyle education.*” – [*MMP*,* Karimnagar].*

*“Yes*,* in every visit*,* I inform patients that hypertension is a chronic disease and that they must take special care in terms of medication*,* diet*,* and exercise for the rest of their lives otherwise it will lead to more complications like heart attack*,* brain stroke*,* paralysis.”–[MMP*,* Karimnagar]*.

One of the AYUSH providers stressed the need for adherence to the changes in lifestyle and medications.

*“I communicate to the patient the risks of hypertension*,* and the probability of getting paralysis*,* heart problem*,* kidney problems*,* etc. if not treated and not adhered to lifestyle changes.”* – [*AYUSH*,* Karimnagar]*.

RMPs customized their advice based on patients’ backgrounds and spent more time counseling than MMPs. Patients often met RMPs informally to discuss health. One RMP detailed how they tailored counseling based on patients’ occupations during home visits.

*“…will give counseling on his food habits*,* and his work. Whether he is a driver*,* or agriculture or ‘hamali’ (manual labour). If he is a driver*,* I tell what they have to do*,* if ‘hamali’ (manual labour) they will bear more load*,* I tell them accordingly like taking water*,* more diet etc. Agricultural worker mostly work in sun. Because of this BP will fluctuate. Hence*,* I suggest them what time to start what time to stop the work.” -[RMP*,* Warangal]*.

“*10–30 Minutes. Sometimes casually discuss with the patients about their health when they come to the clinic for some other problem.”* – [*RMP*,* Karimnagar]*.

Patients confirmed that both MMPs and RMPs helped them understand the importance of lifestyle modification. RMPs also tailored their advice as per the patient’s need.

*“My doctor*,* as well local RMP played a key role in these (lifestyle) modifications. Doctor advised to reduce the salt and RMP doctor told me importance of taking medicine regularly. Earlier I use to take medicine irregularly but after seeing (a person get paralysis due to hypertension)*,* I asked the local RMP(about the person being paralysed)*,* he informed about the adverse effects.” – [42*,* F]*. AYUSH providers counseled patients on hypertension risks, emphasizing exercise, yoga, and reducing salt, alcohol, and tobacco consumption.

### Home-based monitoring

Most of the providers recommended buying a digital BP device for home usage to promote hypertension monitoring and self-management among the patients regularly.

According to an MMP,

*“Yes*,* I recommend patients buy BP devices for home usage. Digital BP monitor is most suitable for home-based.”-[MMP*,* Karimnagar].*

Patient interviews showed varied responses: four of 21 had digital BP devices, others considered buying one, and two didn’t see the need due to a nearby RMP.

*“Yes*,* I have digital monitoring device. But I’m unable to use it” – [73*,* M]*.

“*Not immediately*,* but I am considering buying BP monitor at home.*” – [50,M].

### Implementation of guidelines into practice

All providers mentioned following a hypertension treatment protocol, but did not provide specific details, limiting our ability to assess their knowledge and adherence.

“*Yes*,* I follow the treatment protocol. In Ayurveda there 18 kinds of protocols to examine the patient at primary level like testing of tongue*,* skin*,* eyes*,* nails*,* etc*” – *[Ayurveda*,* Karimnagar].*

“*Follow protocol and guidelines for treating hypertension patients.” –[MMP*,* Siricilla].*

*“I follow protocol provided by the govt.” –[RMP*,* Karimnagar].*

### Distribution/availability of informative material

All providers, formal and informal, educated patients about hypertension, but only two used brochures or leaflets. Some suggested organizing medical camps for hypertension awareness.

“*Educational papers have to be given with figures and images on risk factors*,* consequences*,* comparison like if medicine taken and if not taken…recently I have printed the educational brochure and given to the patients.*” – [RMP, Sircilla].

*“Medical agencies provide leaflets and information brochures. I get them from Medical Representatives and that I use to inform patients.” –[RMP*,* Siricilla]*.

“*Medical camps can be conducted and we can screen the patients*,* give them knowledge.*” – [*RMP*,* Karimnagar].*

### Provider training

MMPs relied on Continuing Medical Education (CME) and WhatsApp groups for information, while RMPs used peer meetings and input from pharmaceutical representatives. Participants called for more frequent CME sessions in rural areas to improve knowledge dissemination. Pharmaceutical sales representatives also played a significant role in providing new information to RMPs.

“*CMEs/RMP meetings as well WhatsApp group and social media. Every month there is a meeting with expert doctors. If a physician comes*,* we clarify our doubts regarding the latest updates on stroke*,* paralysis*,* and nowadays about the coronavirus*, etc.” – *[RMP*,* Karimnagar].*

“*Sources of new knowledge are the latest medical research books*,* CMEs*,* pharma reps*,* WhatsApp*,* etc… There is a need for some information dissemination sessions in the rural area because CMEs are mostly conducted in Hyderabad. Hence focus should be on rural areas.”* – *[MMP*,* Warangal]*.

### Follow-up and adherence

Providers lacked a systematic follow-up system and relied on patients to schedule visits. Recommended follow-up frequencies varied, but adherence was low. Only 20–30% of MMPs’ patients adhered to schedules; others visited when symptomatic.

*“Few will come regularly. Most are irregular. When the problem arises then only they come.” –[MMP*,* Siricilla].*

*“For 1st time they come but after that*,* they come irregularly. Whenever they have symptoms then only they come. It is very common in rural areas.”* – *[RMP*,* Siricilla].*Providers cited reasons for non-adherence to follow-ups: patient unawareness of hypertension risks, tendency to stop treatment after symptoms subside, and financial constraints. Providers mentioned patient’s concerns about continuous drug usage, and high out-of-pocket expenses, which contributed to their non-adherence. RMPs used personal reminders to encourage follow-ups.

“*Due to economic problems and negligence*,* patients discontinue their follow-ups*” – [*RMP*,* Siricilla].*

“*Negligence and not having awareness about the health condition. Some people who have BP at borderline level 150–160*,* think that if they take medicine now*,* they will have to continue it for life. This is also one of the reasons to discontinue the follow-up and medication.”* – *[RMP*,* Karimnagar].*

*“Due to ignorance only*,* they discontinue the follow-ups. Still*,* when they meet me in the village*,* I ask them to come for a BP checkup*,* or if I go for home visits and if they are near*,* I ask them to come and get their BP checked.” –* [*RMP*,* Warangal]*.

Patients also confirmed similar reasons for non-adherence to follow-ups.

*“Doctor said to come regular checkup. Initially*,* I went 2–3 times regularly. When I went for follow up doctor prescribed same medicine and same dosage. I found no problem after using the medicine. Hence*,* I am continuing same medicine for 2 years and not visiting the doctor. I thought if any problem come then I can visit the doctor.”- [46*,* M]*.

*“For B.P I am checking regularly with RMP who is nearby my home. But a Cardiologist I visit once in 6 months. Going to a cardiologist is expensive.” – [43*,* M]*.

*“Every month I get my BP checked at nearby RMP clinic*,* there is no fee but when I go for visit to a MD*,* cardiologist*,* he asks to get tests like 2D echo*,* ECG etc and all that costs around 3000/- to 4000/-” – [63*,* M]*.

### Patient records

MMPs stored patient names, addresses, and contact numbers manually. RMPs, familiar with patients and their community, didn’t maintain records due to perceived lack of value and government recognition. MMPs considered IT solutions for data management, but RMPs lacked resources and perceived no need.

“*No*,* I don’t record any patient’s information. In Chaleur village*,* there are 4 RMPs and each RMP has his patients*,* hence we know the patients’ records.*” – *[RMP*,* Karimnagar]*.

“*We record patient details such as ailments*,* treatment/diagnosis*,* and medication information in a case sheet. The store in charge/receptionist takes this record. When the patient comes again*,* we search for the patient details*” – [*MMP*,* Warangal].*

### Mobilizing community-based resources

RMPs participated in community programs and camps initiated by NGOs or government hospitals, receiving incentives. MMP involvement in such activities was minimal, and none focused on hypertension or non-communicable diseases.

“*Yes*,* I was involved in T.B. camp*,* Health camps*,* Polio*,* etc. Only during Polio drops camp incentive was given.*” – *[RMP*,* Karimnagar]*.

## Discussion

This study provides a comprehensive examination of the entire care pathway for hypertension management in the private healthcare sector in rural and peri-urban areas of Telangana, India. By exploring each element in the chain, from screening to follow-up, and including perspectives from diverse stakeholders, such as RMPs, AYUSH providers, pharmacists, diagnostic labs, and pharmaceutical sales representatives, this study adds new insights to the existing literature on hypertension care in India [26, 27, 31]. The findings not only confirm the presence of challenges identified in other parts of the country but also provide a detailed understanding of the specific barriers and enablers at each step of the care pathway.

First, the study reveals significant gaps in the screening and diagnosis of hypertension among private healthcare providers. Notably, the lack of adherence to guidelines for opportunistic screening and the use of varying diagnostic thresholds and intervals between readings can lead to delayed diagnosis and treatment initiation, increasing the risk of complications and mortality associated with uncontrolled hypertension [26]. The findings highlight that providers often rely on symptoms to initiate screening, rather than following the recommended practice of screening all adults [27]. Furthermore, the study identifies specific factors contributing to this problem, such as the lack of awareness about the importance of opportunistic screening, the use of different blood pressure cut-offs for diagnosis, and the inconsistent intervals between follow-up readings. These findings underscore the need for targeted educational interventions that address the specific knowledge gaps and attitudinal barriers among private healthcare providers, taking into account their diverse backgrounds and practice settings.

Second, moving along the care pathway, the study uncovers the influence of financial incentives on provider behavior and treatment costs [28, 29]. The presence of incentives for referrals and prescriptions, such as commissions from diagnostic labs and pharmaceutical companies, can lead to overdiagnosis, overtreatment, and increased healthcare expenditure [30]. This study provides new evidence on how these incentives operate in the context of hypertension care in rural and peri-urban India, with RMPs receiving incentives for referring patients to MMPs and diagnostic labs, and MMPs being influenced by the performance and incentives associated with specific drugs. These findings emphasize the need for policies and regulations that promote transparency and accountability in the healthcare system, such as mandatory disclosure of financial relationships and the implementation of clinical guidelines for evidence-based prescribing.

Third, continuity of care and patient adherence to treatment are critical elements in the management of hypertension, and this study sheds light on the challenges at this stage of the care pathway. The lack of monitoring systems, poor record-keeping practices, and the absence of a structured follow-up mechanism contribute to low adherence to follow-up visits [31]. The study reveals that providers often place the responsibility of follow-up on the patients, without actively tracking or reminding them of their appointments. Additionally, the findings provide a nuanced understanding of the factors influencing patient behavior, such as high out-of-pocket expenditure, lack of awareness about the consequences of uncontrolled hypertension, and fear of dependence on long-term medication [32]. Patients reported that the cost of consultations, medications, and diagnostic tests posed a significant barrier to seeking care and adhering to treatment. These findings underscore the need for innovative financing mechanisms, such as health insurance schemes and community-based health funds, and the potential of telemedicine and mobile health technologies in facilitating remote monitoring and follow-up, particularly in resource-constrained settings.

The low follow-up rates and non-adherence to guidelines are striking findings of this study. To address these issues, educational efforts targeting both healthcare providers and patients should be prioritized. Provider education should focus on emphasizing the importance of adherence to guidelines, the significance of regular follow-ups, and the use of standardized diagnostic and treatment protocols. This can be achieved through continuing medical education programs, workshops, and the dissemination of evidence-based guidelines through professional networks and associations. Patient education, on the other hand, should aim to increase awareness about the consequences of uncontrolled hypertension, the importance of regular follow-ups, and the benefits of adherence to prescribed treatment. This can be done through community-based health education programs, the distribution of informative materials in local languages, and the use of mass media and social media campaigns.

In addition to educational efforts, the implementation of telehealth and virtual care can play a crucial role in improving patient access to outpatient providers and follow-up on their hypertension management. Telehealth platforms can enable remote consultations, monitoring, and support for patients, particularly those in rural and underserved areas. This can help overcome barriers such as distance, transportation, and time constraints that often hinder patients from seeking care and adhering to follow-up visits. Virtual care tools, such as mobile health applications and remote monitoring devices, can facilitate the tracking of blood pressure, medication adherence, and lifestyle modifications, empowering patients to take an active role in their hypertension management. However, the successful implementation of telehealth and virtual care requires investments in digital infrastructure, training of healthcare providers, and the development of user-friendly and culturally appropriate interfaces.

Patient outreach is another critical aspect of improving hypertension management in the private healthcare sector. Currently, patient outreach efforts at the study sites are limited and often rely on passive approaches, such as waiting for patients to return for follow-up visits. To enhance patient outreach, providers should adopt a more proactive approach, such as regularly reminding patients of their appointments through phone calls, text messages, or home visits by community health workers [33]. Collaborating with local community organizations, religious leaders, and influential figures can help spread awareness and encourage patients to seek care and adhere to treatment. Additionally, establishing community-based screening programs and health camps can help identify undiagnosed cases of hypertension and link them to appropriate care.

Next, patient education and outreach, essential components of the care pathway, are also examined in this study. While providers reported offering lifestyle advice and counseling, the effectiveness of these efforts may be limited by the lack of structured patient education programs and informative materials. The study found that providers often relied on verbal instructions and did not have access to standardized educational resources. This suggests that simply providing information may not be sufficient to change patient behavior and improve self-management practices. The study highlights the need for comprehensive and context-specific patient education interventions that consider the cultural beliefs, health literacy levels, and social determinants of health of the target population. Leveraging local resources, such as RMPs and pharmacies, in delivering patient education and outreach programs can improve the reach and effectiveness of these interventions, as these providers are often more accessible and trusted by the community.

The methodological approach of examining each element in the chain of the care pathway has several notable advantages. Firstly, it allows for a comprehensive and systematic exploration of the various stages of hypertension management, from screening and diagnosis to treatment and follow-up. By doing so, the study can identify specific gaps, barriers, and enablers at each step of the care pathway, providing a more nuanced understanding of the intertwined interplay among provider practices, patient behaviors, and health system factors. Secondly, the inclusion of diverse stakeholders, such as RMPs, AYUSH providers, pharmacists, diagnostic labs, and pharmaceutical sales representatives, enables the study to capture a wide range of perspectives and experiences, thus offering a more holistic view of the challenges and opportunities for improving hypertension care in the private sector.

However, this methodological approach does not come without its challenges and limitations. The purposive sampling technique, while ensuring the inclusion of diverse stakeholders, may introduce selection bias and limit the representativeness of the findings. The focus on a specific geographical region, namely rural and peri-urban areas of Telangana, may restrict the generalizability of the findings to other settings with different socio-economic, cultural, and health system characteristics. Furthermore, the reliance on qualitative data, obtained through in-depth interviews, may be subject to social desirability bias, as participants might provide responses that they perceive to be more acceptable or expected by the researchers. Additionally, the cross-sectional nature of the study does not allow for the examination of temporal changes or causal relationships between the identified factors and hypertension care outcomes. The COVID-19 pandemic also introduced specific challenges. This was particularly notable for MMPs and AYUSH providers, who found themselves with slightly higher patient load, leaving them with limited availability for the interview.

Despite these challenges, the methodological approach employed in this study makes a valuable contribution to the literature on hypertension care in India by providing a comprehensive and context-specific understanding of the challenges and opportunities at each step of the care pathway in the private sector. The findings generated through this approach can serve as a foundation for developing targeted, evidence-based interventions and policies that address the specific barriers and enablers identified at different levels of the healthcare system. However, future research should aim to validate and expand upon these findings using more representative sampling techniques, longitudinal study designs, and mixed-methods approaches to further strengthen the evidence base on improving hypertension care in the private sector in India.

## Conclusion

In conclusion, this study provides valuable insights into the complex challenges of hypertension management in the private healthcare sector in rural and peri-urban areas of India by examining each element in the chain of the care pathway. The findings highlight the need for targeted interventions that address specific barriers and enablers at each stage, such as provider education, innovative financing mechanisms, regulations on financial incentives, strengthening of health information systems, and patient education and outreach programs.

Addressing these challenges will require the concerted efforts of all stakeholders, including policymakers, healthcare providers, civil society organizations, and patients. By prioritizing hypertension control and implementing evidence-based interventions, including educational efforts, telehealth, virtual care, and proactive patient outreach, India can make significant strides in reducing the burden of cardiovascular diseases and improving population health.

Future research should focus on evaluating the effectiveness and cost-effectiveness of specific interventions at each step of the care pathway and exploring their feasibility and acceptability in different settings and populations across India.

## Supplementary Information

Supplementary Material 1.

## Data Availability

The data that support the findings of this study are not publicly available. The data are, however, available from the authors upon reasonable request and with the permission of Max Institute of Healthcare Management, Indian School of Business (MIHM, ISB).

## Abbreviations

CVD: Cardiovascular Diseases
AYUSH: Ayurveda Yoga and Naturopathy Unani Siddha Homeopathy
RMP: Rural Medical Practitioner
NCD: Non–communicable Diseases
MMP: Modern medicine provider
CCM: Chronic Care Model
SBP: Systolic Blood Pressure
DBP: Diastolic Blood Pressure
CME: Continuing Medical Education
