# Supplementary material for: Private provider practices and incentives for hypertension management in rural and peri-urban Telangana, India– a qualitative study
Source: BMC Health Serv Res. 2024 Oct 9;24:1206. doi: 10.1186/s12913-024-11560-5 (PMC11462739; doi:10.1186/s12913-024-11560-5)
Supplement: Supplementary file 1 — Supplementary Material 1. [file 12913_2024_11560_MOESM1_ESM.pdf]

Max Institute of Healthcare Management
Indian School of Business

# Studying the Private Provider Incentives for Effective Management of Hypertension

## Semi-Structured Interview Guide

## General Practitioners (GPs)

*The following information will be recorded prior to the interview:*

| | |
|---|---|
| *Name (practitioner)* | |
| *Age* | |
| *Highest educational qualification* | |
| *Type of medicine being practiced* | |
| *Location of practice* | |
| *Number of years of experience* | |

---

*Purpose of the document: This interview guide represents the main themes to be discussed with participants. It does not necessarily include all the prompts that may be used (however, we have attempted at enlisting the key prompts for each question). Non-leading and general prompts will also be used, such as "Can you please tell me a little bit more about that?" and "What was the outcome?"*

---

## Establishing rapport

1. What are the most common diseases that patients come to you with?
2. Could you tell me about your patient load?
   *Probes:*
      *How many patients visit you every day?*
3. Could you tell me about your patient load?
      *How many hypertensive patients visit you every day?*
      *How many new hypertensive patients do you see every day or in a week?*

## Screening & Diagnosis

1. Do you conduct any screening tests for visiting patients?
   *Probes:*
      *(If yes) Which tests?*
      *(If no) Why not?*
2. What are your judgement criteria for the screening to lead to hypertension diagnosis?
   *Probe:*
      *Which BP measurement device do you use? What is the BP threshold that you consider as high for hypertension?*
      *On what criteria do you decide to do BP test for the patient? Age? If patient reports a particular symptom?*
      *Do you confirm diagnosis on the same day?*
      *Do you use the same day BP reading to confirm hypertension or 2-3 readings spread across say a week?*
3. Which BP device model do you have in your clinic? Is this model recommended for usage at home as well?
4. What actions do you take upon the confirmation of hypertension (with or without additional tests)?
   *Probe:*
      *Do you start the patients on medicine or lifestyle counseling or both?*
      *(if No to medicines) Why do you prefer to not start the patient on medicines right away?*

## Treatment Practices

1. How do you determine which medicines to prescribe to the various strata of hypertensive patients?

2. Do you prefer combination medicines or single molecules?
   *Probe: What is the reason for this preference?*
3. Are there brands that you prefer for hypertension?
   *Probes:*
      *How frequently do you change brands relating to a single molecule?*
      *How frequently do you shift between molecules of the same class of drugs?*

*(For instance, they might have been prescribing Telmisartan back then, but after Olmesartan was introduced, they started prescribing the new molecule- though still an ARB.)*

4. Do you stock medicines at your clinic?
   *Probes:*
   *Which hypertensive medicines do you stock?*

| ***Name*** | ***Strength*** | ***Brand*** |
|---|---|---|
| | | |
| | | |
| | | |
| | | |
| | | |
| | | |
| | | |

*Are any of these medicines provided free (either in the form of sample or in bulk) by the pharma representatives?*

*(If no)*
*Is there a particular reason why you do not stock medicines at your clinic?*

5. Do you suggest any specific pharmacies or do the patients choose as per their will?

## Guideline adherence and knowledge upgradation

1. Do you use a treatment protocol to escalate treatment/titrate treatment?
2. What are your main sources of new knowledge? (CMEs/Pharma reps/WhatsApp groups/others)
   *Probes: Is it efficient to receive info from such a medium? If not, what would you prefer?*

## Data documentation and IT usage

1. Do you record any data about the visiting patients?
   *Probes:*
   *(If yes)*
   *What all do you record?*
   *Who does the recording? (Doctor/Nurse/Receptionist/other)*
   *Where do you store that data?*
   *What is the primary use of collecting the data?*
   *Do you retrieve the records when a patient comes for follow up?*
   *Could you show me a few such records? This is simply for my understanding.*
   *(If no)*
   *Why not?*

2. (If there is no IT system in place) Do you think having an IT system to record the data would help you in any way?
   *Probes:*
   *(If yes)*
   *Do you have any specific IT requirement in mind that might be beneficial?*
   *How would it provide you or your patients any benefit?*
   *Have you considered using simple tracking systems such as SMS, WhatsApp threads with patients?*
   *(If no)*
   *Why not?*

## Follow-up

1. Do you schedule follow-up visits for the patients at specific time intervals, or do you leave it open ended as per their will?
   *Probe: (If the former) According to your advice, how soon should hypertension patients follow-up with you after initial diagnosis?*
2. After the first follow-up, how frequently do you advise controlled and uncontrolled hypertension patients to follow up with you? (or is it upon their will?)
3. What percentage of patients follow-up with you (till they are controlled and after they are controlled)?
4. Do you counsel patients on the importance of follow-ups and harmful risks for non-adherence during each follow-up? *(Probe into their reasoning behind this)*
5. What are the reasons for patients discontinuing their follow-ups?
6. What are the reasons for patients discontinuing their medication?
7. Do you take any measure at your clinic to encourage patients to come back for follow-up visits?
   *Probe:*
   *Who exactly undertakes these actions and what is the typical cost or effort involved in those?*

## Referrals and Incentives

1. How much do you charge a patient for consultation? Is the fee different for first visit and follow-up visits?
2. Do RMPs or AYUSH doctors refer patients to your clinic? Typically, how many patients in a week?
   *Probe:*
   *Do you ask the patients about their interaction with referring RMPs/AYUSH (to assess the practices of these practitioners)?*
   *Prompt: Diagnostic practices, fees, knowledge of diseases*
3. Do RMPs and AYUSH expect any financial consideration in return for the patient referrals? If yes, how much?
4. Do you recommend referred patients to follow up with the initial providers (RMPs/AYUSH)?
   *Probes:*
   *(if Yes)*

*What percentage of patients do you prescribe returning to the initial provider?*
*What are the factors that trigger you to refer patients to the initial providers?*
*What is the recommended follow-up visit frequency with you for these patients?*

*(if No)*

*Do you face any resentment or opposition from the initial providers?*

5. Do you refer any hypertensive patient to other general practitioners or a super-specialist for a secondary consultation?
   *Probes:*
   *What are the most common cases when this referral is done?*
   *What percentage of these patients come back to you after their secondary consultation?*
6. Are there any monetary incentives from a referral by a typical GP to a super-specialist? If yes, how much?
7. Do other GPs expect an incentive for referring their patients to you for a secondary consultation? If yes, how much?
8. Do pharmacies share a part of their revenue with the provider who prescribes those medicines? Approximately what percentage of the revenue? *(Seek separate answers for RMP, AYUSH, GPs, and Super-specialists categories)*
9. Do laboratories share a part of their revenue with the provider who prescribes those tests? Approximately what percentage of the revenue? *(Seek separate answers for RMP, AYUSH, GPs, and Super-specialists categories)*
10. How do pharma companies provide positive reinforcement for doctors that are higher volume prescribers compared to others? Have you experienced that recognition in the past? Can you provide details?

## Understanding practitioner's motivation for hypertension prevention

*(Wherever necessary) For the following questions, probe further to understand factors that can facilitate or hinder implementation of change in practice.*

1. What components of lifestyle management do you counsel the patient on?

| ***Lifestyle component*** | ***Recommendation*** | ***Importance ranking*** |
|---|---|---|
| *Diet* | | |
| *Salt intake* | | |
| *Physical exercise* | | |
| *Alcohol* | | |
| *Tobacco* | | |
| *Other* | | |

2. How much time do you spend on an average on lifestyle education?
3. Could you elaborate on what all do you communicate to the patient regarding the risks of hypertension?
   *Probe:*
   *Do you provide the information or does your staff do so?*

*What risks do you communicate? Do you do that or does your staff do that?*
*Do you have any educational material about hypertension that you give out to the patients?*
*(If yes) Where do you get that from? Have you seen any benefits from its use?*
*(If no) Do you think that providing such material to the patients could be beneficial?*
*Do you inform patients that hypertension is a chronic disease, and that they must take special care (medication, diet, exercise) for the rest of their lives?*
*How do patients react to this information? Do patients value it?*
*Does the knowledge of this affect their follow-up visits? (This probe should be asked in the follow-up section)*
*Do you prefer face to face counseling or providing patients with some audio-visual aid? Why?*

4. Do you recommend patients to buy BP device for home usage? (If yes) Which model?
5. As you are aware, hypertension is asymptomatic in nature. If you are to screen all adults at your clinic, how do you think it could be achieved?
6. What services could be offered through your clinic to improve treatment initiation?
7. What services could be offered through your clinic to improve patient follow-up?
*Probe: In your opinion, is it/would it be useful to call up patients to remind them of an upcoming follow-up or prescription refills?*
8. In the past, have you ever been a part of any government initiatives to improve public health programmes (such as immunization, maternal & child health, etc)?
*Probe:*
*(if Yes)*
*Was there any incentive?*
*Who approached you for providing services in the camp?*
*How was your experience with those programme?*

## HR and operational roles of staff members

1. Who all works in your clinic? Could you describe the HR structure and operational roles of your staff members?
2. What are their individual roles and responsibilities?
3. What are their educational qualifications?
4. How do you and your staff keep yourself updated about newly introduced procedures and guidelines? Where do you receive such information from?
5. Do they have a smartphone?
6. Which member in your staff contacts patients for keeping track of them and for nudging them to follow-up?
7. Is there a possibility of clinic staff doing screening of all patients while they are in the waiting area? What are your views on this suggestion?
8. Can the staff call the patients to remind them of follow up visits? What are your views on this suggestion?

## Concluding the interview

Switch off the digital recorder.

Thank you for your time and much-valued inputs to the study!

Is there anything else that you would like to add to what has been discussed?

(She/he answers)

Again, thanks for your inputs. As mentioned earlier, we'll use these insights to understand the motivations of private providers to provide hypertension care, and we'll then outline prospective interventions to improve their engagement for managing hypertension better. Also, all responses will be kept confidential; your de-identified interview responses will only be shared with research team members and we will ensure that any information we include in our report does not identify you as the respondent.

Ask permission to get back to interviewee for any clarifications/further information.

I look forward to staying in touch with you and will keep you updated on our study!

# Studying the Private Provider Incentives for Effective Management of Hypertension

## Semi-Structured Interview Guide

## Rural medical practitioners (RMPs)

*The following information will be recorded prior to the interview:*

| | |
|---|---|
| *Name (practitioner)* | |
| *Age* | |
| *Highest educational qualification* | |
| *Type of medicine being practiced* | |
| *Location of practice* | |
| *Number of years of experience* | |

---

*Purpose of the document: This interview guide represents the main themes to be discussed with participants. It does not necessarily include all the prompts that may be used (however, we have attempted at enlisting the key prompts for each question). Non-leading and general prompts will also be used, such as "Can you please tell me a little bit more about that?" and "What was the outcome?"*

---

## Establishing rapport

1. What are the most common diseases that are reported in this area?
2. Could you tell me about your patient load?
   *Probes:*
   *How many patients visit you every day?*
3. Could you tell me about your patient load?
   *How many hypertensive patients visit you every day?*
   *How many new hypertensive patients do you see every day or in a week?*

## Screening & Diagnosis

1. Do you conduct any screening tests for visiting patients?
   *Probes:*
   *(If yes) Which tests?*
   *(If no) Why not?*
2. What are your judgement criteria for the screening to lead to hypertension diagnosis?
   *Probe:*
   *Which BP measurement device do you use? What is the BP threshold that you consider as high for hypertension?*
   *On what criteria do you decide to do BP test for the patient? Age? If patient reports a particular symptom?*
   *Do you confirm diagnosis on the same day?*
   *Do you use the same day BP reading to confirm hypertension or 2-3 readings spread across say a week?*
3. Which BP device model do you have in your clinic? Is this model recommended for usage at home as well?
4. What actions do you take upon the confirmation of hypertension (with or without additional tests)?
   *Probe:*
   *Do you start the patients on medicine or lifestyle counseling or both?*
   *(if No to medicines) Why do you prefer to not start the patient on medicines right away?*

## Treatment Practices

1. How do you determine which medicines to prescribe to the various strata of hypertensive patients?
2. Are there brands that you prefer for hypertension? Why?
3. Do you stock medicines at your clinic?
   *Probes:*
   *Which hypertensive medicines do you stock?*

| ***Name*** | ***Strength*** | ***Brand*** |
|---|---|---|
| | | |
| | | |
| | | |

| | | |
|---|---|---|
| | | |
| | | |
| | | |
| | | |

*Are any of these medicines provided free (either in the form of sample or in bulk) by the pharma representatives?*

*(If no)*

*Is there a particular reason why you do not stock medicines at your clinic?*

4. Do you suggest any specific pharmacies or do the patients choose as per their will?

## Guideline adherence and knowledge upgradation

1. Do you use a treatment protocol to escalate treatment/titrate treatment?
2. What are your main sources of new knowledge? (CMEs/Pharma reps/WhatsApp groups/others)
   *Probes: Is it efficient to receive info from such a medium? If not, what would you prefer?*

## Data documentation and IT usage

1. Do you record any data about the visiting patients?
   *Probes:*
   *(If yes)*
   *What all do you record?*
   *Who does the recording? (Doctor/Nurse/Receptionist/other)*
   *Where do you store that data?*
   *What is the primary use of collecting the data?*
   *Do you retrieve the records when a patient comes for follow up?*
   *Could you show me a few such records? This is simply for my understanding.*
   *(If no)*
   *Why not?*
2. (If there is no IT system in place) Do you think having an IT system to record the data would help you in any way?
   *Probes:*
   *(If yes)*
   *Do you have any specific IT requirement in mind that might be beneficial?*
   *How would it provide you or your patients any benefit?*
   *Have you considered using simple tracking systems such as SMS, WhatsApp threads with patients?*
   *(If no)*
   *Why not?*

## Follow-up

1. Do you schedule follow-up visits for the patients at specific time intervals, or do you leave it open ended as per their will?

*Probe: (If the former) According to your advice, how soon should hypertension patients follow-up with you after initial diagnosis?*

2. After the first follow-up, how frequently do you advise controlled and uncontrolled hypertension patients to follow up with you? (or is it upon their will?)
3. What percentage of patients follow-up with you (till they are controlled and after they are controlled)?
4. Do you counsel patients on the importance of follow-ups and harmful risks for non-adherence during each follow-up? *(Probe into their reasoning behind this)*
5. What are the reasons for patients discontinuing their follow-ups?
6. What are the reasons for patients discontinuing their medication?
7. Do you take any measure at your clinic to encourage patients to come back for follow-up visits?
   *Probe:*
      *Who exactly undertakes these actions and what is the typical cost or effort involved in those?*

## Referrals and Incentives

1. How much do you charge a patient for consultation? Is the fee different for first visit and follow-up visits?
2. Do other RMPs refer patients to your clinic? Typically, how many patients in a week?
   *Probe:*
      *Do you ask the patients about their interaction with referring RMPs (to assess the practices of these practitioners)?*
      *Prompt: Diagnostic practices, fees, knowledge of diseases*
3. Do the referring RMPs expect any financial consideration in return for the patient referrals? If yes, how much?
4. Do you recommend referred patients to follow up with the initial providers (RMPs)?
   *Probes:*
      *(if Yes)*
         *What percentage of patients do you prescribe returning to the initial provider?*
         *What are the factors that trigger you to refer patients to the initial providers?*
         *What is the recommended follow-up visit frequency with you for these patients?*
      *(if No)*
         *Do you face any resentment or opposition from the initial providers?*
5. Do you refer any hypertensive patient to general practitioners or a super-specialist for a secondary consultation?
   *Probes:*
      *What are the most common cases when this referral is done?*
      *What percentage of these patients come back to you after their secondary consultation?*
6. Are there any monetary incentives from a referral by a typical RMP to a GP? If yes, how much?
7. Do other RMPs expect an incentive for referring their patients to you for a secondary consultation? If yes, how much?

8. Do pharmacies share a part of their revenue with the provider who prescribes those medicines? Approximately what percentage of the revenue? *(Seek separate answers for RMP, AYUSH, GPs, and Super-specialists categories)*
9. Do laboratories share a part of their revenue with the provider who prescribes those tests? Approximately what percentage of the revenue? *(Seek separate answers for RMP, AYUSH, GPs, and Super-specialists categories)*
10. How do pharma companies provide positive reinforcement for doctors that are higher volume prescribers compared to others? Have you experienced that recognition in the past? Can you provide details?

## Understanding practitioner's motivation for hypertension prevention

*(Wherever necessary) For the following questions, probe further to understand factors that can facilitate or hinder implementation of change in practice.*

1. What components of lifestyle management do you counsel the patient on?

| ***Lifestyle component*** | ***Recommendation*** | ***Importance ranking*** |
|---|---|---|
| *Diet* | | |
| *Salt intake* | | |
| *Physical exercise* | | |
| *Alcohol* | | |
| *Tobacco* | | |
| *Other* | | |

2. How much time do you spend on an average on lifestyle education?
3. Could you elaborate on what all do you communicate to the patient regarding the risks of hypertension?
   *Probe:*
   *What risks do you communicate? Do you do that or do you have a staff that does that?*
   *Do you have any educational material about hypertension that you give out to the patients?*
   *(If yes) Where do you get that from? Have you seen any benefits from its use?*
   *(If no) Do you think that providing such material to the patients could be beneficial?*
   *Do you inform patients that hypertension is a chronic disease, and that they must take special care (medication, diet, exercise) for the rest of their lives?*
   *How do patients react to this information? Do patients value it?*
   *Does the knowledge of this affect their follow-up visits? (This probe should be asked in the follow-up section)*
   *Do you prefer face to face counseling or providing patients with some audio-visual aid? Why?*
4. Do you recommend patients to buy BP device for home usage? (If yes) Which model?
5. As you are aware, hypertension is asymptomatic in nature. If you are to screen all adults at your clinic, how do you think it could be achieved?
6. What services could be offered through your clinic to improve treatment initiation?

7. What services could be offered through your clinic to improve patient follow-up?
   *Probe: In your opinion, is it/would it be useful to call up patients to remind them of an upcoming follow-up or prescription refills?*
8. In the past, have you ever been a part of any government initiatives to improve public health programmes (such as immunization, maternal & child health, etc)?
   *Probe:*
   *(if Yes)*
   *Was there any incentive?*
   *Who approached you for providing services in the camp?*
   *How was your experience with those programme?*

## HR and operational roles of staff members

1. Who all works in your clinic?
2. What are their individual roles and responsibilities?
3. What are their educational qualifications?
4. How do you and your staff keep yourself updated about newly introduced procedures and guidelines? Where do you receive such information from?
5. Do they have a smartphone?
6. Which member in your staff contacts patients for keeping track of them and for nudging them to follow-up?
7. Is there a possibility of clinic staff doing screening of all patients while they are in the waiting area? What are your views on this suggestion?
8. Can the staff call the patients to remind them of follow up visits? What are your views on this suggestion?

## Concluding the interview

Switch off the digital recorder.

Thank you for your time and much-valued inputs to the study!

Is there anything else that you would like to add to what has been discussed?

(She/he answers)

Again, thanks for your inputs. As mentioned earlier, we'll use these insights to understand the motivations of private providers to provide hypertension care, and we'll then outline prospective interventions to improve their engagement for managing hypertension better. Also, all responses will be kept confidential; your de-identified interview responses will only be shared with research team members and we will ensure that any information we include in our report does not identify you as the respondent.

Ask permission to get back to interviewee for any clarifications/further information.

I look forward to staying in touch with you and will keep you updated on our study!

# Studying the Private Provider Incentives for Effective Management of Hypertension

## Semi-Structured Interview Guide

## AYUSH practitioners

*The following information will be recorded prior to the interview:*

| | |
|---|---|
| *Name (practitioner)* | |
| *Age* | |
| *Highest educational qualification* | |
| *Type of medicine being practiced* | |
| *Location of practice* | |
| *Number of years of experience* | |

---

*Purpose of the document: This interview guide represents the main themes to be discussed with the participants. It does not necessarily include all the prompts that may be used (however, we have attempted at enlisting the key prompts for each question). Non-leading and general prompts will also be used, such as "Can you please tell me a little bit more about that?" and "What was the outcome?"*

---

## Establishing rapport

1. What are the most common diseases that are reported in this area?
2. Could you tell me about your patient load?
   *Probes:*
   *How many patients visit you every day?*
3. Could you tell me about your patient load?
   *How many hypertensive patients visit you every day?*
   *How many new hypertensive patients do you see every day or in a week?*

## Screening & Diagnosis

1. Do you conduct any screening tests for visiting patients?
   *Probes:*
   *(If yes) Which tests?*
   *(If no) Why not?*
2. What are your judgement criteria for the screening to lead to hypertension diagnosis?
   *Probe:*
   *Which BP measurement device do you use? What is the BP threshold that you consider as high for hypertension?*
   *On what criteria do you decide to do BP test for the patient? Age? If patient reports a particular symptom?*
   *Do you confirm diagnosis on the same day?*
   *Do you use the same day BP reading to confirm hypertension or 2-3 readings spread across say a week?*
3. Which BP device model do you have in your clinic? Is this model recommended for usage at home as well?
4. What actions do you take upon the confirmation of hypertension (with or without additional tests)?
   *Probe:*
   *Do you start the patients on medicine or lifestyle counseling or both?*
   *(if No to medicines) Why do you prefer to not start the patient on medicines right away?*

## Treatment Practices

1. How do you determine which medicines to prescribe to the various strata of hypertensive patients?
2. Are there brands that you prefer for hypertension? Why?
3. Do you stock medicines at your clinic?
   *Probes:*
   *Which hypertensive medicines do you stock?*

| ***Name*** | ***Strength*** | ***Brand*** |
|---|---|---|
| | | |
| | | |
| | | |

| | | |
|---|---|---|
| | | |
| | | |
| | | |
| | | |

*Are any of these medicines provided free (either in the form of sample or in bulk) by the pharma representatives?*

*(If no)*

*Is there a particular reason why you do not stock medicines at your clinic?*

4. Do you suggest any specific pharmacies or do the patients choose as per their will?

## Guideline adherence and knowledge upgradation

1. Do you use a treatment protocol to escalate treatment/titrate treatment?
2. What are your main sources of new knowledge? (CMEs/Pharma reps/WhatsApp groups/others)
   *Probes: Is it efficient to receive info from such a medium? If not, what would you prefer?*

## Data documentation and IT usage

1. Do you record any data about the visiting patients?
   *Probes:*
   *(If yes)*
   *What all do you record?*
   *Who does the recording? (Doctor/Nurse/Receptionist/other)*
   *Where do you store that data?*
   *What is the primary use of collecting the data?*
   *Do you retrieve the records when a patient comes for follow up?*
   *Could you show me a few such records? This is simply for my understanding.*
   *(If no)*
   *Why not?*
2. (If there is no IT system in place) Do you think having an IT system to record the data would help you in any way?
   *Probes:*
   *(If yes)*
   *Do you have any specific IT requirement in mind that might be beneficial?*
   *How would it provide you or your patients any benefit?*
   *Have you considered using simple tracking systems such as SMS, WhatsApp threads with patients?*
   *(If no)*
   *Why not?*

## Follow-up

1. Do you schedule follow-up visits for the patients at specific time intervals, or do you leave it open ended as per their will?

*Probe: (If the former) According to your advice, how soon should hypertension patients follow-up with you after initial diagnosis?*

2. After the first follow-up, how frequently do you advise controlled and uncontrolled hypertension patients to follow up with you? (or is it upon their will?)
3. What percentage of patients follow-up with you (till they are controlled and after they are controlled)?
4. Do you counsel patients on the importance of follow-ups and harmful risks for non-adherence during each follow-up? *(Probe into their reasoning behind this)*
5. What are the reasons for patients discontinuing their follow-ups?
6. What are the reasons for patients discontinuing their medication?
7. Do you take any measure at your clinic to encourage patients to come back for follow-up visits?

   *Probe:*

   *Who exactly undertakes these actions and what is the typical cost or effort involved in those?*

## Referrals and Incentives

1. How much do you charge a patient for consultation? Is the fee different for first visit and follow-up visits?
2. Do other AYUSH or RMPs refer patients to your clinic? Typically, how many patients in a week?

   *Probe:*

   *Do you ask the patients about their interaction with referring RMPs/AYUSH (to assess the practices of these practitioners)?*

   *Prompt: Diagnostic practices, fees, knowledge of diseases*
3. Do the referring RMPs/AYUSH expect any financial consideration in return for the patient referrals? If yes, how much?
4. Do you recommend referred patients to follow up with the initial providers (RMPs/AYUSH)?

   *Probes:*

   *(if Yes)*

   *What percentage of patients do you prescribe returning to the initial provider?*

   *What are the factors that trigger you to refer patients to the initial providers?*

   *What is the recommended follow-up visit frequency with you for these patients?*

   *(if No)*

   *Do you face any resentment or opposition from the initial providers?*
5. Do you refer any hypertensive patient to general practitioners or super-specialists for a secondary consultation?

   *Probes:*

   *What are the most common cases when this referral is done?*

   *What percentage of these patients come back to you after their secondary consultation?*
6. Are there any monetary incentives from a referral by a typical AYUSH to a GP? If yes, how much?

7. Do other AYUSH or RMPs expect an incentive for referring their patients to you for a secondary consultation? If yes, how much?
8. Do pharmacies share a part of their revenue with the provider who prescribes those medicines? Approximately what percentage of the revenue? *(Seek separate answers for RMP, AYUSH, GPs, and Super-specialists categories)*
9. Do laboratories share a part of their revenue with the provider who prescribes those tests? Approximately what percentage of the revenue? *(Seek separate answers for RMP, AYUSH, GPs, and Super-specialists categories)*
10. How do pharma companies provide positive reinforcement for doctors that are higher volume prescribers compared to others? Have you experienced that recognition in the past? Can you provide details?

## Understanding practitioner's motivation for hypertension prevention

*(Wherever necessary) For the following questions, probe further to understand factors that can facilitate or hinder implementation of change in practice.*

1. What components of lifestyle management do you counsel the patient on?

| ***Lifestyle component*** | ***Recommendation*** | ***Importance ranking*** |
|---|---|---|
| *Diet* | | |
| *Salt intake* | | |
| *Physical exercise* | | |
| *Alcohol* | | |
| *Tobacco* | | |
| *Other* | | |

2. How much time do you spend on an average on lifestyle education?
3. Could you elaborate on what all do you communicate to the patient regarding the risks of hypertension?
   *Probe:*
   - *What risks do you communicate? Do you do that or do you have a staff that does that?*
   - *Do you have any educational material about hypertension that you give out to the patients?*
     - *(If yes) Where do you get that from? Have you seen any benefits from its use?*
     - *(If no) Do you think that providing such material to the patients could be beneficial?*
   - *Do you inform patients that hypertension is a chronic disease, and that they must take special care (medication, diet, exercise) for the rest of their lives?*
     - *How do patients react to this information? Do patients value it?*
     - *Does the knowledge of this affect their follow-up visits? (This probe should be asked in the follow-up section)*
   - *Do you prefer face to face counseling or providing patients with some audio-visual aid? Why?*
4. Do you recommend patients to buy BP device for home usage? (If yes) Which model?

5. As you are aware, hypertension is asymptomatic in nature. If you are to screen all adults at your clinic, how do you think it could be achieved?
6. What services could be offered through your clinic to improve treatment initiation?
7. What services could be offered through your clinic to improve patient follow-up?
   *Probe: In your opinion, is it/would it be useful to call up patients to remind them of an upcoming follow-up or prescription refills?*
8. In the past, have you ever been a part of any government initiatives to improve public health programmes (such as immunization, maternal & child health, etc)?
   *Probe:*
   *(if Yes)*
   *Was there any incentive?*
   *Who approached you for providing services in the camp?*
   *How was your experience with those programme?*

## HR and operational roles of staff members

1. Who all works in your clinic?
2. What are their individual roles and responsibilities?
3. What are their educational qualifications?
4. How do you and your staff keep yourself updated about newly introduced procedures and guidelines? Where do you receive such information from?
5. Do they have a smartphone?
6. Which member in your staff contacts patients for keeping track of them and for nudging them to follow-up?
7. Is there a possibility of clinic staff doing screening of all patients while they are in the waiting area? What are your views on this suggestion?
8. Can the staff call the patients to remind them of follow up visits? What are your views on this suggestion?

## Concluding the interview

Switch off the digital recorder.

Thank you for your time and much-valued inputs to the study!

Is there anything else that you would like to add to what has been discussed?

(She/he answers)

Again, thanks for your inputs. As mentioned earlier, we'll use these insights to understand the motivations of private providers to provide hypertension care, and we'll then outline prospective interventions to improve their engagement for managing hypertension better. Also, all responses will be kept confidential; your de-identified interview responses will only be shared with research team members and we will ensure that any information we include in our report does not identify you as the respondent.

Ask permission to get back to interviewee for any clarifications/further information.

Max Institute of Healthcare Management
Indian School of Business

I look forward to staying in touch with you and will keep you updated on our study!

# Studying the Private Provider Incentives for Effective Management of Hypertension

## Semi-Structured Interview Guide

## Pharmacies

*The following information will be recorded prior to the interview:*

| | |
|---|---|
| *Name of participant* | |
| *Gender* | |
| *Age* | |
| *Educational Qualification* | |
| *Role in the pharmacy* | |
| *What is your daily patient load?* | |
| *Number of days the pharmacy is open for in a week:* | |
| *Timing of the pharmacy:* | |
| *Location of practice:* | |
| *Are there any large hospitals/clinics nearby?* | |

---

*Please note that this guide only represents the main themes to be discussed with the participants and as such does not necessarily include all the prompts that may be used (however, we have attempted at enlisting the key prompts for each question). Non-leading and general prompts will also be used, such as "Can you please tell me a little bit more about that?" and "What was the outcome?"*

---

## Building rapport

1. How many patients visit your pharmacy on a typical day? Could you tell me about their general profile (age, economic background, gender, etc.)?
2. What are the fast-moving items in your pharmacy?

## Understanding the outline of their business model

1. How do you decide what brands of medicines to keep?
   *Probes:*
   *How do factors, such as pharmaceutical representatives, doctors' preferences, pharmacist's interests, and paying capacity of people in the area affect which brand of medicine (and whether generic medicines) are ordered?*
2. Are you supposed to publish your selling price of medicines in a public display?
3. Do you engage in home-delivery model?
   *Probes: (If yes) Why do you do so? How are such requests placed? How are home-deliveries coordinated?*
4. How does your business get affected by new online pharmacies and neighborhood pharmacies?
5. Do patients carry prescription for antihypertensive medicines? What about other medicines (say antibiotics, etc.)?
6. What kind of hypertension medicines are prescribed by doctors, AYUSH and RMPs? Is there a difference by drug class, molecule and/or brand?
7. Do doctors prescribe formulation or brands? Does this behavior hold true for AYUSH and RMPs?

## Referral structure

1. How do you establish/sustain relations with doctors in this area?
2. Do doctors in this area refer their patients to specific pharmacies, or do they leave it up to the patient's choice?
   *Probe:*
   *(If it is the latter) What are the key factors that affect a patient's choice of a particular pharmacy?*
   *What factors affect the purchase of a particular drug and brand?*
3. Of your daily patient load, what proportion come on their own?
   *Probes:*
   *Please provide the break-down of the above figure for RMPs, AYUSH, GPs, and super-specialists.*
   *Do laboratories refer patients to your pharmacy?*
4. Do patients tend to consult you directly for medication without consult a doctor?
   *Probe:*
   *(If yes) Is directly visiting a pharmacy quite common in this area?*
5. Do you provide incentives to medical practitioners for referrals?

*Probes:*

*(If yes) Could you share some information on the incentives?*
*Do you refer patients to doctors? (And/or) If yes Do doctors give any gifts to you for referrals?*
*Do doctors refer patients to you? If yes, do you give any gifts to the doctors for these referrals?*
*(And/or)*
*How do doctors track the purchase made at your pharmacy?*

6. Do pharma companies provide positive reinforcement to pharmacies that are higher volume sellers compared to others? Have you experienced that recognition in the past? Can you provide details?
   *Probe:*
   *What discounts or offers do pharma companies offer to you to stock their medicines?*
   *Do they give you any incentives for above-average sales of their brand in this area?*
7. Do you know if doctors get gifts from pharma companies to prescribe their medicines?
8. Do you know if laboratories give gifts to doctors to prescribe diagnostic tests?

## Hypertension care

1. What proportion of daily sales at your pharmacy are for hypertension?
2. How frequently do hypertensive patients follow-up to purchase medicines from your pharmacy?
3. Which medicines (and brands) are prescribed by doctors the most?
   *Probe: Do MBBS, AYUSH, etc. prescribe different brands? Which companies do they prefer?*

| ***Name*** | ***Strength*** | ***Brand*** | ***Provider category (AYUSH/GP/etc.)*** |
|---|---|---|---|
| | | | |
| | | | |
| | | | |
| | | | |
| | | | |
| | | | |
| | | | |

4. What are your typical profit margins on commonly sold hypertension medicines? Could you compare that with the margins for the medicines for diabetes and cough/cold?

| ***Name*** | ***Strength*** | ***Brand*** | ***Discount offered to patients*** | ***Profit Margins*** | ***Comparison of this margin with diabetes and cough/cold*** |
|---|---|---|---|---|---|
| | | | | | |
| | | | | | |
| | | | | | |
| | | | | | |

| | | | | | |
|---|---|---|---|---|---|
| | | | | | |
| | | | | | |
| | | | | | |

5. What percentage of hypertensive patients buy all the prescribed medicines?
   *Probe:*
      *If they ask you for your opinion, what do they typically ask, and what is your response?*
      *What is the usual duration for which patients buy medicines? (Prompt: 7 days, 14 days, 30 days). Would you know what determines this decision?*
6. How much does a hypertensive patient typically spend on one purchase?
7. Does your pharmacy engage in any diagnostic test for hypertension?
   *Probes:*
      *(If yes)*
         *What are the tests that you conduct?*
         *What are the measurements in these tests that you consider as a threshold for labelling the patient as hypertensive?*
         *How much do you charge for each test?*
         *How much does it cost you to for conducting each test?*
         *What is the time taken for each of these tests?*
8. Is there a tendency for the patients to consult you for symptomatic treatment instead of doctors for the follow-ups?
9. (If yes) Do you measure BP? If no, who in your staff measures BP, and which medicines do you sell in such cases?
10. Do you sell BP devices?
    *Probes:*
       *Which type and brand?*
       *What are the profit margins on these devices?*
       *Have you received any complaints from the customers, or have there been any issues with selling these devices? Please elaborate.*
11. Do you or someone from the pharmacy follow-up with chronic care patients for medicine refills?
    *Probe:*
       *(If yes) How do you connect with the patient? (WhatsApp / Call / SMS / others)*
       *(If no) Do you think this would be useful for your business?*
12. Do you offer any annual package or discounts, especially to chronic care patients?

## Data documentation and IT usage

1. What all data are you supposed to record as per regulatory compliance?
2. Do you record any data about visiting patients?
   *Probes:*
      *(If yes)*
         *What all do you record?*
         *How do you record that data?*
         *Where do you store that data?*

*What is the primary use of collecting the data?*
*Could you show me a few such records? This is simply for my understanding.*
*(If no)*
*Why not?*

3. (If there is no IT system in place) Do you think having an IT system to record the data would help you in any way?
   *Probes:*
   *(If yes)*
   *How?*
   *(If no)*
   *Why not?*

## Staff

1. Could you share with me the details of your staff?
   *Probes:*
   *Who all works in your pharmacy?*
   *What are their individual roles and responsibilities?*
   *What are their salaries?*
   *Are their work timings the same as yours?*
   *What are their educational qualifications? Yours?*
2. How do you and your staff keep yourself updated about new medicines in the market? Where do you receive such information from?
3. Do you / does your staff use a smartphone? Which communication apps do you use on a day-to-day basis?

## Concluding the interview

Switch off the digital recorder.

Thank you for your time and much-valued inputs to the study!

Is there anything else that you would like to add to what has been discussed?

(She/he answers)

Again, thanks for your inputs. As mentioned earlier, we'll use these insights to understand the motivations of private providers to provide hypertension care, and we'll then outline prospective interventions to improve their engagement for managing hypertension better.

Ask permission to get back to the interviewee for any clarifications/further information.

I look forward to staying in touch with you and will keep you updated on our study!

Max Institute of Healthcare Management
Indian School of Business

# Studying the Private Provider Incentives for Effective Management of Hypertension

## Semi-Structured Interview Guide

## Pharmaceutical Sales Representatives

*The following information will be recorded prior to the interview:*

| | |
|---|---|
| *Participant Name* | |
| *Company name* | |
| *Designation* | |
| *Educational Qualification* | |

---

*Please note that this guide only represents the main themes to be discussed with the participants and as such does not necessarily include all the prompts that may be used (however, we have attempted at enlisting the key prompts for each question). Non-leading and general prompts will also be used, such as "Can you please tell me a little bit more about that?" and "What was the outcome?"*

---

## Building rapport

1. Which geographical area are you in charge of? Are you the only representative appointed by your company to manage this area?
2. How does a typical working day look like for you?
   *Probe:*
   *How many pharmacies do you typically visit on a given day?*
   *How many doctors do you typically meet on a given day?*
   *Which categories of doctors do you typically engage with (RMPs, AYUSH, GPs, Super-specialists)?*
   *Do you also engage with any laboratories? (If yes) What are the reasons for doing so?*
3. How do your monthly targets look like? Who in your company sets them for you?
4. What are the fast-moving medicines in your catalogue?

## Engagement with doctors

1. Do doctors in this area refer their patients to specific pharmacies, or do they leave it up to the patient's choice?
   *Probe:*
   *(If it is the latter) What are the key factors that affect a patient's choice of a particular pharmacy?*
   *What factors affect the purchase of a particular drug and brand?*
2. Which anti-hypertensives do GPs, AYUSH and RMPs prescribe the most? Is there a difference by drug class, molecule and/or brand?

| ***Name*** | ***Strength*** | ***Brand*** | ***Provider category (AYUSH/GP/etc.)*** |
|---|---|---|---|
| | | | |
| | | | |
| | | | |
| | | | |
| | | | |
| | | | |
| | | | |

   *Probe:*
   *Do GPs and Super-specialists prescribe formulation or brands? Does this behavior hold true for AYUSH and RMPs?*
   *For how many days do doctors typically prescribe anti-hypertensives?*
3. How do you establish/sustain relations with doctors in this area?
   *Probe:*
   *How frequently do you visit them?*
   *How does a typical conversation sound like?*

4. How do you incentivize the doctors in your area to prescribe your brand over others? How do other brands' representatives incentivize these doctors?
5. What are the key factors that influence a doctor's choice in prescribing a specific brand?

## Engagement with pharmacies

1. How do pharmacies decide which brands of medicines to keep?
   *Probes:*
   *How do factors, such as pharmaceutical representatives, doctors' preferences, pharmacist's interests, and paying capacity of people in the area affect which brand of medicine (and whether generic medicines) are ordered?*
2. What are the various anti-hypertensives that your company sells? What's the state-wide best performer (in terms of monthly sales)?
   *Probe: Is the trend the same in the geographic area that you cater to?*
3. How do your anti-hypertensives perform as compared to the sales figures of your most-sold medicines? Also, how do they compare against sales of medication for diabetes?
4. What is the typical order quantity and order frequency for anti-hypertensives at pharmacies?
   *Probe: How does that compare with medications for commonly sold antibiotics and medicines for diabetes and cough/cold?*
5. How do pharmacies place new orders with your brand?
   *Probe: Is there a centralized IT system that you enroll them on, or is it via phone calls to you on an ad-hoc basis?*
6. What is the proportion of the volume of your anti-hypertensives sold as compared to the total daily sales of all your products in any pharmacy?
7. What are your typical profit margins on your anti-hypertensives? Could you compare that with the margins for commonly sold antibiotics and medicines for diabetes and cough/cold?

| ***Name*** | ***Strength*** | ***Discount offered to pharmacies*** | ***Profit Margins*** | ***Comparison of this margin with commonly sold antibiotics and medicines for diabetes and cough/cold*** |
|---|---|---|---|---|
| | | | | |
| | | | | |
| | | | | |
| | | | | |
| | | | | |
| | | | | |
| | | | | |

8. What are typical profit margins on these for the pharmacies? Could you again compare that with the margins for pharmacies on commonly sold antibiotics and medicines for diabetes and cough/cold?
9. Have you faced scenarios in which pharmacies reach stockout point for any of your products (demand greater than the supply leading to zero availability)?
   *Probes:*

*For which product(s)?*
*What are the factors that lead to stockout? (If the pharmacies could have anticipated such a scenario, they would have had adjusted the order quantity/frequency beforehand itself, which is not the case here)*
*How do you respond to such situations?*
*Has it ever occurred for any anti-hypertensive? Which one?*

10. Is your selling price flexible with respect to your relationship with pharmacies?
    *Probe:*
    *(if yes)*
    *Which factors influence your decision-making about the selling price point?*
    *Is there a variation for anti-hypertensives too?*
    *(if no)*
    *Is that a mandate according to your company's policies?*
11. What positive reinforcements do you provide to pharmacies that are higher volume sellers compared to others? Can you provide details?
    *Probe: Do you offer any annual packages or discounts to your high-volume pharmacies, especially for anti-hypertensive purchases?*

## Concluding questions

1. What all data do you typically record in your day-to-day activities?
2. How do keep yourself updated about new medicines of competitive brands in the market? Where do you receive such information from?

## Concluding the interview

Switch off the digital recorder.

Thank you for your time and much-valued inputs to the study!

Is there anything else that you would like to add to what has been discussed?

(She/he answers)

Again, thanks for your inputs. As mentioned earlier, we'll use these insights to understand the motivations of private providers to provide hypertension care, and we'll then outline prospective interventions to improve their engagement for managing hypertension better.

Ask permission to get back to the interviewee for any clarifications/further information.

I look forward to staying in touch with you and will keep you updated on our study!

# Studying the Private Provider Incentives for Effective Management of Hypertension

## Semi-Structured Interview Guide

## Laboratories

*The following information will be recorded prior to the interview:*

| | |
|---|---|
| *Name of participant* | |
| *Gender* | |
| *Age* | |
| *Educational Qualification* | |
| *Role in the laboratory* | |
| *What is your daily patient load?* | |
| *Number of days the lab is open for in a week:* | |
| *Timing of the lab:* | |
| *Location of practice:* | |
| *Are there any large hospitals/clinics nearby?* | |

---

*Please note that this guide only represents the main themes to be discussed with the participants and as such does not necessarily include all the prompts that may be used (however, we have attempted at enlisting the key prompts for each question). Non-leading and general prompts will also be used, such as "Can you please tell me a little bit more about that?" and "What was the outcome?"*

---

## Establishing Rapport

1. What are the various tasks that you do on a daily basis in the lab?
2. What are the common diagnostic tests that are done in your laboratory on a day to day basis?
3. How many patients visit your laboratory on a typical day? Could you tell me about their general profile (age, economic background, gender, etc.)?

## General demand and supply dynamics, and an overview of their business model

1. Where are your collection centers located? How are these locations chosen?
2. Are you supposed to publish the prices of tests in a public display?
3. Do you also dispense medicines at your laboratory for any disease?
   *Probes:*
   *(If yes)*
   *(probe deep with focus on hypertension)*
   *For what all diseases?*
   *Which brands?*
   *How many patients do you sell medicines to on a weekly basis?*
   *Where do you procure them from?*
   *What are the unit profit margins on them?*

## Referral structure

1. How do you establish/sustain relations with doctors and pharmacies in this area?
2. How do people typically get to know about your laboratory?
   *Probes: Referral from practitioners / patient's search for a laboratory near his home / others*
3. Do doctors in this area refer their patients to specific laboratories or do they leave it up to the patient's choice?
   *Probe: (If it is the latter) What are the key factors that affect a patient's choice of a particular laboratory?*
4. What fraction of visitors to your laboratory are referred by doctors?
   *Probes: What is the proportion of patients referred by each of the three categories: RMPs, GPs, and super-specialists?*
5. Do pharmacies refer patients to your laboratory?
6. Why do the directly-visiting patients not consult a doctor before visiting your laboratory? Is directly visiting a laboratory quite common in this area?
7. Is it a common practice for patients to carry their prescription to get diagnostic tests?
   *Probe:*
   *(If yes) What proportion of patients carry prescription?*
   *(If no) Why don't patients carry prescription?*
8. Could you share some information on the incentives that laboratories have to provide to medical practitioners for referrals?
   *Probes:*
   *What are the various tie-ups that you have with RMPs, GPs, and super-specialists?*

*What are their expectations for a patient referral?*
*How do they track the tests done at your laboratory?*

9. How do equipment manufacturers provide positive reinforcement to laboratories? Have you experienced that recognition in the past? Can you provide details?
   *Probes:*
   *Who exactly is provided the incentive? (Lab technician/owner/etc.)*
10. Are there any incentives on reagents and consumables?

## Hypertension care

1. Do you know what common tests are prescribed by doctors to diagnose hypertension?

| ***Test*** | ***Purpose*** | ***Cost to consumer*** | ***Price to your lab*** | ***Time taken to conduct*** | ***Threshold measurement for hypertension labelling*** | ***Is staff trained to conduct this test?*** | ***Do doctors expect a cut?*** |
|---|---|---|---|---|---|---|---|
| | | | | | | | |
| | | | | | | | |
| | | | | | | | |
| | | | | | | | |
| | | | | | | | |
| | | | | | | | |
| | | | | | | | |

*(If they say there are no specific tests for hypertension) What are the tests that you conduct for chronic diseases such as hypertension and diabetes? Is there some sort of a package?*

2. How much is the profit margin for these tests? How does it compare to that for the tests for other common diseases such as diabetes?
3. How frequently do hypertensive patients get follow-ups tests conducted? Which tests?
   *Note: If we do not get specific information on tests, then (a) consider interviewing pathologist and not administrative person, and (b) use list of tests that doctors mention that they prescribe and ask about those specifically.*
4. Which equipment do you typically use for hypertension tests?
5. What are the hurdles in delivery of samples? Do you face any hindrance in the maintenance of smooth cold chain for blood work?
6. What according to you are the problems faced by hypertensive patients in terms of receiving care service?
   *Probes:*
   *Do you think your customers would appreciate a free BP check at your establishment?*
   *Do you have a customer loyalty programme for chronic care patients?*
   *Does your lab send any reminder SMSs or calls to patients to come back for regular check-ups?*

## Data documentation and IT usage

1. What all data are you supposed to record as per regulatory compliance?

2. Do you record any data about the visiting patients?
   *Probes:*
   *(If yes)*
   *What all do you record?*
   *How do you record that data?*
   *Where do you store that data?*
   *What is the primary use of collecting the data?*
   *Could you show me a few such records? This is simply for my understanding.*
   *(If no)*
   *Why not?*
3. (If there is no IT system in place) Do you think having an IT system to record the data would help you in any way?
   *Probes:*
   *(If yes)*
   *How?*
   *(If no)*
   *Why not?*

## Staff

1. Could you share with me the details of your staff?
   *Probes:*
   *Who all works in your laboratory?*
   *What are their individual roles and responsibilities?*
   *What is the total staff strength in this lab? (Split between technical and support staff)*
   *What are their salaries?*
   *Are their work timings the same as yours?*
   *What are their educational qualifications? Yours?*
2. How do you and your staff keep yourself updated about newly introduced procedures and tests? Where do you receive such information from?
3. Do you / does your staff use a smartphone? Which communication apps do you use on a day-to-day basis?

## Concluding the interview

Switch off the digital recorder.

Thank you for your time and much-valued inputs to the study!

Is there anything else that you would like to add to what has been discussed?

(She/he answers)

Again, thanks for your inputs. As mentioned earlier, we'll use these insights to understand the motivations of private providers to provide hypertension care, and we'll then outline prospective interventions to improve their engagement for managing hypertension better. Also, all responses will be kept confidential; your de-identified interview responses will only be shared with research team members and we will ensure that any information we include in our report does not identify you as the respondent.

Ask permission to get back to interviewee for any clarifications/further information.

I look forward to staying in touch with you and will keep you updated on our study!

# Studying the Private Provider Incentives for Effective Management of Hypertension

## Semi-Structured Interview Guide

## Patients

## Introduction

*The following information will be recorded prior to the interview:*

| | |
|---|---|
| *Age* | |
| *Sex* | |
| *Occupation* | |
| *Income per year* | |
| *Educational qualification* | |
| *Information on family (who all lives at home)* | |
| *How long have you been living in this area?* | |
| *Where did you live before shifting to this area? What was your occupation then?* | |

---

*Please note that this guide only represents the main themes to be discussed with the participants and as such does not necessarily include all the prompts that may be used (however, we have attempted at enlisting the key prompts for each question). Non-leading and general prompts will also be used, such as "Can you please tell me a little bit more about that?" and "What was the outcome?"*

---

## Hypertension screening and diagnosis

1. When did you find out you had hypertension?
2. Where did you find that out (At a camp / at a doctor's clinic / etc.)?
3. Were there any particular symptoms at that time? Or did you visit the doctor for some other ailment but found that you had hypertension?
   *Probe: Since how long were you experiencing those symptoms? Why did you not visit a doctor sooner?*
4. Did you ask to get your BP checked or did the provider check it for you?
5. After how many BP readings did the doctor confirm that you are hypertensive?
   *Probe: Were these readings taken the same day?*
6. What was your BP during screening?
7. Whom did you first consult (RMP/GP/AYUSH/Super-specialist/pharmacy/others)? What was the first-visit consultation fee?
   *Probe: (In case of pharmacy or RMP or AYUSH) Why did you visit that particular category of doctor?*
8. What other lab tests did the doctor prescribe/conduct?

| ***Test*** | ***Cost*** | ***Where was it conducted?*** |
|---|---|---|
| | | |
| | | |
| | | |
| | | |
| | | |
| | | |

9. (In case the screening tests took place in a laboratory) Was a specific lab recommended to you by your doctor?
   *Probes:*
   *(If yes)*
   *Could you describe your visit to the lab?*
   *(If no)*
   *Which lab did you choose? What were the factors that led you to choose that lab?*
10. Did your doctor refer you to some other doctor (such as a specialist)?
    *Probe: (If yes) Why did the doctor refer to you a specialist? How much did the specialist charge?*
11. Which medicines were you started on?

| ***Name*** | ***Strength*** | ***Brand*** | ***Cost*** |
|---|---|---|---|
| | | | |
| | | | |

| | | | |
|---|---|---|---|
| | | | |
| | | | |
| | | | |

12. Where did you purchase your medicines from? What factors influenced your choice?

## Self-management of hypertension by the patient

1. Can you describe what you do on a daily basis to manage hypertension?
   *Probes: What changes have you made in your diet? Exercise routine?*
   *What was your primary motivation?*
   *How big a role did your doctor play in these modifications?*
2. How do you keep track of your BP?
   *Probes:*
   *How frequently do you need to check your BP as suggested by your doctor?*
   *Do you check your BP regularly as prescribed? Where (and how) do you measure it or get it measured?*
   *Have you had instances when your BP measured abruptly high or low? Could you tell more about those instances and how did you manage them?*
   *Have you considered buying a home BP monitoring device?*
3. Can you share some details about your medicine consumption behavior?
   *Probes:*
   *Which medicines are you currently consuming? What's the prescribed dosage?*
   *Have there been instances when you had forgotten to consume your medicines?*
   *Have you had instances when you decided not to consume?*
   *Have you had instances when you self-altered the dosage?*
   *Has your medication changed since you were started on hypertensive medications?*
   *(If yes)*
   *What were you previously consuming?*
   *Why the change?*
   *Did the doctor ask you to change those medicines? If not, who advised and why?*
   *(If they are having non-certified medicines) Why are you not consuming allopathic medicines?*

## Patient's interaction with the healthcare system

1. What is the follow-up consultation fee charged by your doctor?
   *Probe: Does your doctor offer you any kind of annual/semi-annual package, since hypertension is likely to be a lifelong problem? (If no) Do you think that such a package might be useful for you?*
2. How often are you asked to follow-up?
   *Probe:*

*Are you reminded by your doctor when it is time to follow-up? How does he stay in touch with you?*

3. Are you able to follow-up with the doctor regularly?
   *Probes:*
   *(If no)*
   *Why not? (probe deeper into whether it is due to symptomatic relief)*
4. How does your doctor keep track of your visits?
5. Can you seek advice from your provider on WhatsApp / over a call / SMS or do you have to visit him/her in-person?
6. How often did the previous doctor ask you to come to his clinic when you were diagnosed with hypertension?
   *Probe: How often did you go? When did you stop going? Why did you stop?*
7. How much money do you typically spend every month on hypertension treatment?
   *Probe:*
   *Could you provide a breakdown of this expenditure? (Consultation, medicines, travel, loss of wages etc.).*
   *Do you have additional expenditure due to a changed diet?*
   *What duration do you usually purchase medicines for?*

## Hypertension-awareness

1. How much time does the doctor spend with you? Were you able to get answers to all your questions?
2. Is your doctor motivated to give you additional time for hypertension-related educational purposes?
   *Probes:*
   *Were you told that hypertension does not have any symptoms?*
   *Were you informed on lifestyle changes and diet improvement to control hypertension?*
   *Were you explained the short-term and long-term effects of hypertension if left uncontrolled? What were you told?*
3. In your opinion, how relevant is the information that was shared with you by your doctor or you?
   *Probe:*
   *Has your doctor given you additional information on hypertension management, would it have had any influence on you or your course of treatment?*
   *What do you think are the most important points that hypertensive patients should ideally be informed about by their doctors?*

## Concluding the interview

1. Have I missed out on asking you about any other challenges that you faced during the course of your hypertension medication?

2. Do you have any suggestions for doctors, pharmacies, or laboratories for improving hypertension care?

Switch off the digital recorder.

Thank you for your time and much-valued inputs to the study!

Is there anything else that you would like to add to what has been discussed?

(She/he answers)

Again, thanks for your inputs. As mentioned earlier, we'll use these insights to understand the motivations of private providers to provide hypertension care, and we'll then outline prospective interventions to improve their engagement for managing hypertension better. Also, all responses will be kept confidential; your de-identified interview responses will only be shared with research team members and we will ensure that any information we include in our report does not identify you as the respondent.

Ask permission to get back to interviewee for any clarifications/further information.

I look forward to staying in touch with you and will keep you updated on our study!
